# Closing the gap on causal processes of infection risk from cross-sectional data: structural equation models to understand infection and co-infection

**DOI:** 10.1186/s13071-015-1274-7

**Published:** 2015-12-23

**Authors:** Scott Carver, Julia A. Beatty, Ryan M. Troyer, Rachel L. Harris, Kathryn Stutzman-Rodriguez, Vanessa R. Barrs, Cathy C. Chan, Séverine Tasker, Michael R. Lappin, Sue VandeWoude

**Affiliations:** School of Biological Sciences, University of Tasmania, Hobart, TAS Australia; Faculty of Veterinary Science, University of Sydney, Sydney, NSW Australia; Department of Biomedical Sciences, Oregon State University, Corvallis, OR USA; Department of Microbiology, Immunology, and Pathology, Colorado State University, Fort Collins, CO USA; The Animal Doctors Pte Ltd., Singapore, Singapore; School of Veterinary Sciences, University of Bristol, Langford, Bristol UK; Department of Clinical Sciences, Colorado State University, Fort Collins, CO USA

## Abstract

**Background:**

Epidemiological studies of disease exposure risk are frequently based on observational, cross-sectional data, and use statistical approaches as crucial tools for formalising causal processes and making predictions of exposure risks. However, an acknowledged limitation of traditional models is that the inferred relationships are correlational, cannot easily distinguish direct from indirect determinants of disease risk, and are often considerable simplifications of complex interrelationships. This may be particularly important when attempting to infer causality in patterns of co-infection through pathogen-facilitation.

**Methods:**

We describe analyses of cross-sectional data using structural equation models (SEMs), a contemporary advancement on traditional regression approaches, based on our study system of feline gammaherpesvirus (FcaGHV1) in domestic cats.

**Results:**

SEMs strongly supported a latent (host phenotype) variable associated with FcaGHV1 exposure and co-infection risk, suggesting these individuals are simply more likely to become infected with multiple pathogens. However, indications of pathogen-covariance (potential facilitation) were also variably detected: potentially among FcaGHV1, *Bartonella* spp and *Mycoplasma* spp.

**Conclusions:**

Our models suggest multiple exposures are primarily driven by host phenotypic traits, such as aggressive male phenotypes, and secondarily by pathogen-pathogen interactions. The results of this study demonstrate the application of SEMs to understanding epidemiological processes using observational data, and could be used more widely as a complementary tool to understand complex cross-sectional information in a wide variety of disciplines.

## Background

An important goal of epidemiological research is to identify the causal processes driving observed spatial and temporal patterns of disease in complex environments. Such studies are often based on observational, cross-sectional data (e.g. individual characteristics, disease status, environmental parameters), which may then be used to understand ecological interactions between parasites, host species and the environment. Beyond understanding the driving mechanisms behind disease, a crucial application of this information is to make predictions about disease risk and exposure, thereby informing disease prevention and supporting future experiments [1–4]. Statistical approaches are invaluable tools for formalising causal processes and making predictions of disease exposure risks. For example, models such as logistic regression or risk factor analyses are often used to identify individual host or environmental characteristics associated with increased infection risk (e.g. age, sex, immune status [5–7]). However, an acknowledged limitation of such causative models is that the inferred relationships are over-simplified, often largely correlational [3, 8, 9], and do not necessarily identify direct determinants of disease risk. For example, it may be that predictors such as sex or age do not directly *cause* infection or exposure with a pathogen, but are associated with underlying behavioral or physiological host phenotypes that drive infection risk, such as animal dispersal, territoriality, aggression, sexual contacts and immune status [4]. Additionally, infection or exposure status with one pathogen may facilitate infection with another pathogen (pathogen-facilitation) [2, 10, 11]. However, results from regression analyses cannot easily distinguish between relationships due to pathogen-facilitation, or other factors, such as underlying behaviors that result in some individuals being likely to be infected by multiple pathogens simultaneously. Consequently, the relative importance of individual host characteristics, pathogen-facilitation and their interactions is often poorly understood. Since cross-sectional studies are central to epidemiological investigations, statistical approaches that help overcome these limitations of traditional approaches (and are relatively straightforward to perform and understand) are valuable.

We describe analyses of cross-sectional epidemiological data using structural equation models (SEMs [8, 12]). SEMs involve the development and assessment of theoretical models based on a pre-conceived conceptual framework, and can be used to model complex, multivariate relationships among variables [9, 13]. SEMs derive from statistical techniques including path analysis, simultaneous equation models and factor analysis, but are a contemporary advancement on traditional regression approaches, because they enable models to be specified in a more mechanistic, flexible framework, including direct and indirect relationships among predictor variables [8, 13, 14]. Importantly, SEMs can include ‘latent variables’, which are designed to reflect factors that are not directly observable, but are identified either directly or indirectly by other measured variables [9, 12, 13]. SEMs are particularly well suited to cross-sectional studies of determinants of individual exposure and co-infection, because they allow specification of underlying unmeasured (latent) causes of infection, and for covariance of pathogen infection (co-infection) to be simultaneously accounted for [8, 9, 13]. For example, latent variables can represent ‘host phenotypes’ , which are identified by other measured factors, such as sex or age (which are not themselves proximate causes of exposure, but contribute to the overall host phenotype which does influence exposure risk [15]). Similarly, where important relationships may exist but their directionality cannot be known *a priori*, these can be modelled as covariance relationships; such as is the case for examining co-infection information from ‘snap-shot’ cross-sectional data, which may represent cases of pathogen facilitation (i.e., one pathogen influencing the susceptibility of a host to be infected with another).

The use of SEM (and other related causal analysis approaches) is rapidly expanding in epidemiology [9, 16]. Emerging areas particularly include occupational exposure assessment [17], multiple determinants of single disease exposures [18, 19], behavioral studies linked to disease [20], environmental determinants of disease [21], and non-transmissible diseases [22]. The application of SEM to study co-infection remains rare [23–25], but can be used to examine relationships among measured variables (pathogens) without requiring *a priori* knowledge of pathogen exposure order.

Here, we demonstrate the use of structural equation modelling to extend from risk factor analyses and gain greater mechanistic insight into the determinants of infection, utilising data from our study system on feline gammaherpesvirus (FcaGHV1) infection in domestic cats [26, 27]. Our study is an advancement on previous SEM-based epidemiological research which has either (a) considered direct effects of sex and age, but not treated these as contributing to a latent host phenotype variable (e.g., [25]), or (b) not considered co-infecting pathogens as possible determinants of one another (e.g., [24]), or (c) considered pathogen effects on one another, but without *a priori* knowledge of the order of infection (many non SEM studies, e.g., [7]), for which our study does not make this assumption. We have previously used risk factor analyses to show that being older and male significantly contribute to increased FcaGHV1 infection risk [26]. Our earlier analyses also suggest FcaGHV1 infection is associated with poor health or infection with several co-pathogens [26]. We hypothesise that the observed associations of FcaGHV1infection status with other pathogens may derive from individuals, such as older males, that display host phenotypes (e.g., behavioral, physiological) with greater inherent risk for exposure to multiple pathogens. The causes of these pathogen-pathogen associations could also be a form of pathogen-facilitation, whereby exposure to one pathogen predisposes a host to increased susceptibility to infection with another pathogen, such as via immunosuppression or antibody-dependent enhancement [1, 7, 10]. These causal processes, host phenotype and pathogen-facilitation, may not be mutually exclusive, and we model them simultaneously within an SEM framework.

## Methods

We examined observational data on feline gammaherpesvirus (FcaGHV1) infection prevalence in domestic cats in the United States, Singapore and Australia, coupled with predictors of FcaGHV1 infection status: sex, age and infection status with other pathogens [26]. For all countries, data on individual infection status with other potential co-pathogens included serology for *Bartonella* spp IgG, and PCR assays for *Bartonella clarridgeiae*, *Bartonella henselae*, *Mycoplasma haemofelis* (Mhf) and ‘*Candidatus* Mycoplasma haemominutum’ (Mhm). For Singapore and USA, additional data on *Toxoplasma gondii* IgG and feline immunodeficiency virus (FIV) antibody serology were available. For Singapore only, feline leukaemia virus (FeLV) status (from PCR assay) was also included in the analyses.

We constructed SEMs that reflected the two hypothesised mechanisms driving FcaGHV1 infection status in domestic cats: (1) an underlying host phenotype, and (2) pathogen-facilitation. We modelled the host phenotype as a latent variable that could be predicted by individual sex and age, and predicted FcaGHV1 or other pathogen statuses. The inclusion of a latent host phenotype variable with contributory sex and age observed variables is a highly plausible framework to specify in relation to pathways of multiple pathogen exposure. To estimate pathogen interrelationships, including potential pathogen-facilitation, we included pathogen-pathogen covariance in the model. Variables to include in SEMs were guided by the significant (*P <*0.05) and near-significant (*P* < 0.2) outcomes of the binomial regression models described by Beatty et al. [26]. In preliminary analyses, we also evaluated whether inclusion of other non-significant predictors of FcaGHV1 improved model fit (as assessed by Akaike’s Information Criterion corrected for small sample sizes, AICc [28]), but this was never the case. We followed [13, 29], and more recent package advancements available through lavaan (www.lavaan.ugent.be) to check alignment with SEM assumptions. Model fit was assessed using a chi-square statistic, and additionally scrutinized using a root mean square error of approximation (RMSEA) and a comparative fit index (CFI), as recommended by [13]. We used a diagonally weighted least squares SEM estimator method, which is appropriate for endogenous categorical variables [13, 29]. We included FcaGHV1 infection status as fixed against our latent variable for model specification purposes. We present non-standardised coefficients and covariances (as standardised covariances are infinite against the fixed FcaGHV1 variable), and acknowledge that this precludes direct comparison among coefficient effect sizes [13, 29], which we are careful to avoid.

Pathogen-pathogen covariance is not restricted to FcaGHV1with other pathogens (see Spearman correlation, Table 1), so we also evaluated all possible combinations of pathogen covariance within the best fit SEMs; again evaluating if inclusion of pathogen covariance improved model fit at each step. For the sake of clarity of study findings, we present only the most parsimonious SEM models for all countries combined and for each country independently. All analyses were undertaken in the program *R* version 3.1.0 [30] using the *stats* [30], *ltm* [31] and *lavaan* [29] packages.

**Table 1:** Pathogen co-infection status is strongly correlated in domestic cats

FIV Ab			Combined			0.131			USA				
*T*. Ab						−0.051	−0.041						
*B*. Ab	0.119**					0.206**	0.059		−0.070				
Bhen	−0.010	0.120**				0.040	0.106		−0.036	0.065			
Bclar	0.042	0.169****	0.052			0.149	0.106		−0.027	0.137	−0.099		
Mhm	0.350****	0.056	0.057	0.037		0.372****	0.090		−0.075	0.159	−0.149	0.118	
Mhf	0.276****	0.031	−0.007	0.089*	0.268****	0.240**	−0.044		−0.033	0.047	−0.018	0.171*	0.300***
FIV Ab			Australia			0.364****			Singapore				
FeLV						0.249****	0.204***						
*T*. Ab						0.018	0.106	−0.015					
*B*. Ab	0.127					−0.042	−0.053	−0.078	0.003				
*Bhen*	−0.143	0.092				−0.035	−0.049	−0.043	−0.026	−0.038			
*Bclar*	−0.102	0.133	0.166			−0.035	−0.049	0.120	−0.026	0.139*	−0.012		
Mhm	0.213*	−0.090	0.280***	−0.129		0.364****	0.206***	0.264***	0.088	−0.121	−0.038	−0.038	
Mhf	0.299***	0.127	−0.043	−0.030	0.235**	0.313****	0.015	0.035	−0.042	−0.061	−0.019	−0.019	0.166**
	FcaGHV1	*B.* Ab	Bhen	Bclar	Mhm	FcaGHV1	FIV Ab	FeLV	*T*. Ab	*B*. Ab	Bhen	Bclar	Mhm

Values represent Spearman correlation *ρ*-values with significant or near significant correlations in bold and their level denoted by *. *P*-values: * 0.05-0.10, ** 0.01-0.05, *** 0.001-0.01, **** < 0.001. All pathogens form chronic infections and thus serology (antibody, Ab) is also a marker of infection; although infection in some seropositive individuals may be latent. In all cases, predictor variables are binomial (0 or 1), with 1 equal to male, adult, or positive serological or infection status. *Abbreviations: B* *Bartonella* spp; *Bclar* Bartonella clarridgeiae, *Bhen* Bartonella henselae, *FcaGHV1* feline gammaherpesvirus 1, *FeLV* feline leukaemia virus, *FIV* feline immunodeficiency virus, *Mhf* Mycoplasma haemofelis, *Mhm* Candidatus Mycoplasma haemominutum, *T* Toxoplasma gondii. Table reproduced from [26Table S2].

### Ethics approval

Results presented here are derived from data presented in Beatty et al. [26]. In that study, samples were collected according to the University of Sydney Animal Ethics Committee approvals (N00/6-2009/1/4985; N00/7-2013/3/6029), Section 53 of the Animals and Birds Act (Cap 5) Agri-Food and Veterinary Authority of Singapore and the Colorado State University Animal Care and Use Committee or appropriate institutional, local, and state agencies.

## Results

**Fig. 1:**
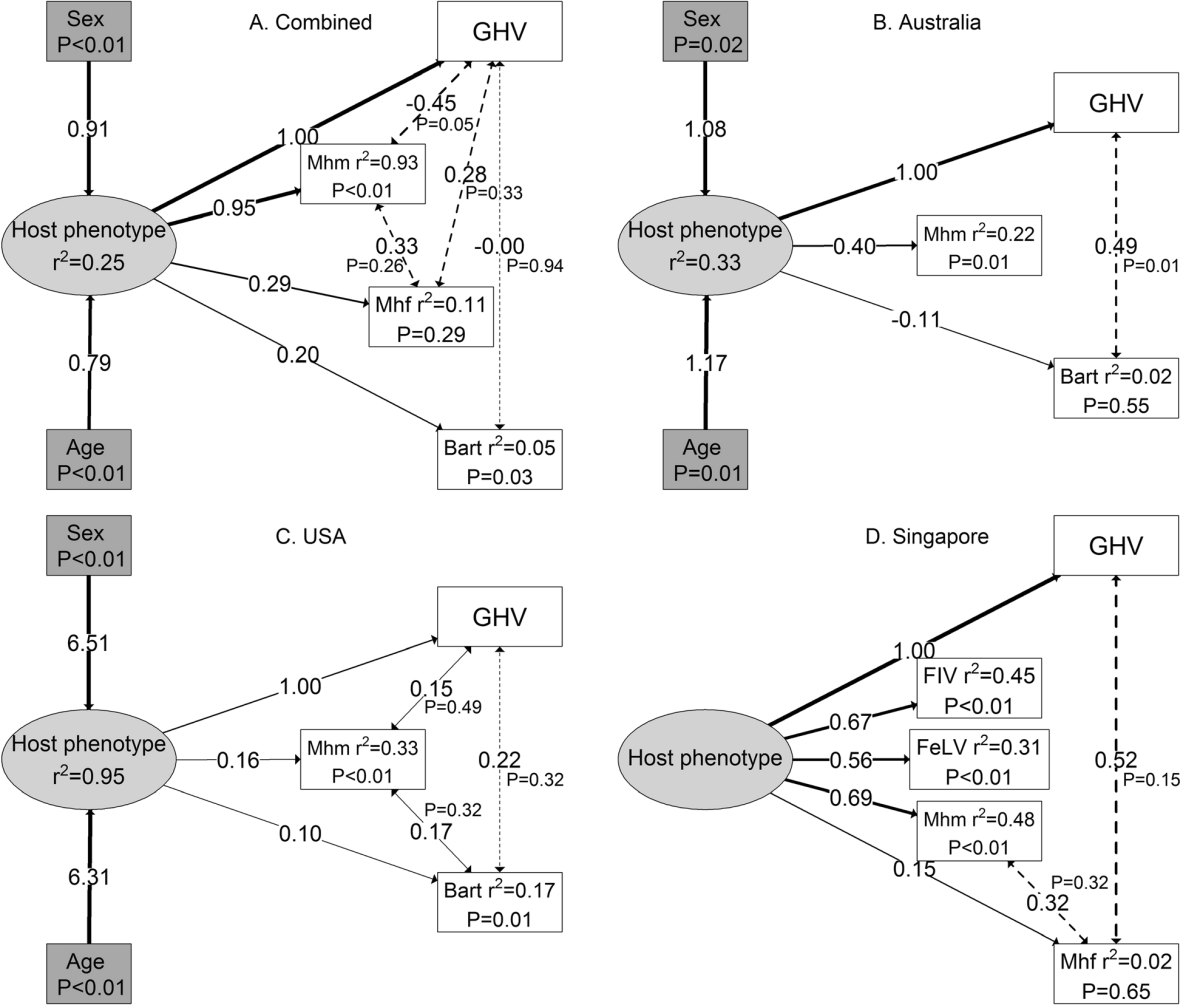
Optimal SEMs showing host phenotype as a latent variable predicting disease co-infection in domestic cats: (a) all sites combined, (b) Australia, (c) USA, (c) Singapore. Boxes and ellipses represent measured and latent variables respectively. Coefficient of determination (r^2^) given where variables are predicted. *P*-values given for measured variables which are predicted, except for FcaGHV1 (abbreviated here to GHV for editorial purposes) which was set as the fixed variable (i.e., a direct response of host phenotype). Solid lines represent directional relationships, with non-standardised coefficients on each line. Dotted lines represent covariance relationships among pathogens (pathogen-facilitation), with their non-standardised coefficients. Line thicknesses are proportional to the strength of coefficient or covariance. In all cases, variables are binomial (0 or 1), with 1 equal to male, adult, or positive pathogen status. Since coefficients are non-standardised, care must be taken not to make direct comparisons of their effect sizes. Abbreviations: Bart: *Bartonella* spp;GHV: FcaGHV1, feline gammaherpesvirus 1; FeLV: feline leukaemia virus; FIV: feline immunodeficiency virus; Mhf: *Mycoplasma haemofelis*; Mhm: *Candidatus* Mycoplasma haemominutum

Structural equation models strongly supported evidence for a latent variable, in this case host phenotype, associated with FcaGHV1, and other pathogen, exposure risk (Fig. 1). For all countries combined (Fig. 1a) the latent variable was significantly predicted by increasing age and being male, positively predicted FcaGHV1, Mhm, *Bartonella* spp, and weakly predicted Mhf infection status. Variables with ‘weak’ effects were identified by their non-significant *P*-values and low r^2^ values, but were still present where they contributed to model parsimony. There was some variation in model structure among countries. The best SEM model for Australia (Fig. 1b) was broadly similar to the overall model, but Mhf infection status was not predicted by host phenotype, likely owing to only a single positive individual (1/84), and *Bartonella* spp was only weakly predicted by host phenotype. Similarly, in the USA (Fig. 1c) the latent variable positively predicted FcaGHV1, *Bartonella* spp and Mhm statuses, but not Mhf infection status. The SEM model for Singapore (Fig. 1d) also provided support for the latent host phenotype variable, which positively predicted FcaGHV1 and Mhm status, and weakly predicted Mhf status. However, a key difference was that unlike Australia, the USA or the overall model, the latent variable in the Singapore model was not predicted by sex and age. The latent variable also positively predicted FIV and FeLV infection status in Singapore, but there was no covariance of infection with these viruses with FcaGHV1or Mycoplasma infection statuses.

Evidence of pathogen covariance was common among our most parsimonious models. All best fit SEM models included pathogen covariance of FcaGHV1 with at least one other pathogen, and most included covariance between other pathogens. For all countries combined (Fig. 1a), the most parsimonious SEM included pathogen covariance of FcaGHV1 with Mhm, Mhf and *Bartonella* spp, and Mhf with Mhm. In Australia (Fig. 1b), FcaGHV1 covaried with *Bartonella* spp infection status, but not with Mhm infection. The best fit SEM for USA (Fig. 1c) included covariance of FcaGHV1, Mhm and *Bartonella* spp infection statuses. In Singapore (Fig. 1d), there was evidence for covariance of FcaGHV1 with Mhf, and Mhm with Mhf infection status. Interestingly, the best fit SEMs were not improved by covariance of immunosuppressive retroviruses (FIV in Singapore and USA, and FeLV in Singapore) with any other pathogen.

## Discussion

We used structural equation modelling to move beyond the correlational nature of results from the risk factor analysis, and generate a more mechanistic interpretation of whether observed patterns of infection were associated with an underlying latent host phenotype (e.g. behavioral or physiological factors that cause older males to be more prone to infection) or covariance among pathogens (pathogen-facilitation). Our results are broadly similar to our original risk factor analysis study [26], but there are several key advances which emphasise the usefulness of SEMs as a complementary tool to more traditional regression techniques.

This study provides strong supporting evidence for the latent variable (host phenotype) to predict FcaGHV1 infection risk in domestic cats: the latent variable significantly and positively predicted FcaGHV1 infection in all four SEM models presented here. Overall, our results suggest that the latent host phenotype is likely the major driver of multiple pathogen exposures. This is predicated on three important points: (1) direct path coefficients from host phenotype to pathogen exposures were more frequently statistically significant than pathogen covariance relationships; (2) the SEM models that we present (which represent the most parsimonious structure) generally contain fewer pathogen covariance relationships than what are possible; and (3) there are multiple cases of host phenotype determining individual pathogen exposure without covariance, but no cases of pathogen covariance without contributory host phenotypic factors. While it appears clear in our models that the latent host phenotype is the dominant determinant of multiple exposures, it is important to recognise that pathogen covariance contributed to model parsimony in all cases, suggesting that there is still a potential (lesser) role of pathogen facilitation on multiple exposures. Thus, we conclude that our models suggest multiple exposures are primarily driven by host phenotypic traits, such as aggressive male phenotypes, and secondarily driven by pathogen-pathogen interactions.

In three of the four SEMs, the latent variable was significantly predicted by sex and age, strongly suggesting FcaGHV1 infection risk in domestic cats is at least partly driven by a behavioral or physiological phenotype associated with being an older male (beyond simply being older and male as implied by our previous risk factor analysis [26]). However, sex and age did not significantly contribute to the latent variable in Singapore, suggesting a host phenotype is a significant predictor of disease risk, but is associated with individual or population characteristics not related to sex or age. Singapore is a densely-populated urban environment, and underlying regional differences such as population density, competition and aggression in both sexes [26] could contribute to host phenotype and therefore disease risk. Combined, these results suggest that a latent host behavioral or physiological phenotype, often associated with older male cats, is likely the major driver of multiple pathogen exposures. Put simply, some individuals are more likely to become exposed to greater numbers of pathogens in their lifetimes. This result has analogy to ecological theory suggesting that a proportion of the host population (older males in our case) experiences a disproportionately large role in exposure and co-infection events. This may in turn suggest contact heterogeneity in the populations, and that some individuals have a disproportionately large role in transmission events [32]. Our study may suggest a link between risk of one and multiple pathogens relating to host phenotype, namely hosts engaging in risky behaviors.

Importantly, the SEM for Singapore suggests the latent variable (not associated with sex or age) predicts not only FcaGHV1, but also FIV, FeLV, Mhm and (to a lesser extent) Mhf infection. FcaGHV1, FIV and FeLV infection status are strongly correlated, and our previous risk factor analyses suggested FIV and FeLV infection were associated with FcaGHV1 infection [26]. Here, the Singapore SEM indicates that FcaGHV1 infection risk is not directly predicted by FIV or FeLV infection status – instead, infection risk of all three viruses is predicted by the latent variable. This result highlights an important distinction: immunosuppressive agents may influence disease progression of pathogens, but these effects may be unrelated to exposure.

The fit of our most parsimonious SEMs were improved by including pathogen covariance in all cases, indicating pathogen-facilitation may be common and contributes to multiple exposures (although the specific pathogens involved varied between models). Information regarding the pathology and epidemiology of these organisms is evolving, thus highlighting an area for more research targeting mechanisms of pathogen-facilitation. Pathogen covariance involves complex networks of interspecific interactions and differential effects among pathogen and host immune responses, which is the subject of much contemporary research [10, 11, 33]. While our analyses suggest pathogen covariance relationships are important, we acknowledge there are limitations to their interpretation, since like traditional regression approaches, SEMs cannot infer the order of pathogen infection from ‘snap-shot’ cross-sectional studies, and therefore the direction of any pathogen facilitation. This directionality can only be overcome by longitudinal sampling or experimentation. Nonetheless, because a considerable number of epidemiological studies are undertaken in a cross-sectional manner, it is useful to consider analytical approaches that accommodate that as best as possible, which we feel our study contributes to. In our study, pathogen covariance evaluates if measured variables are related, but without known directionality (as distinct from specifying causal paths, which implies causation). By specifying the relationship among pathogens as covariance, we take a conservative approach to examining pathogen-pathogen relationships.

## Conclusions

The results of this study demonstrate the application of SEMs to understanding epidemiological processes using cross-sectional research as a complementary tool to other, more traditional analyses. Our study makes advances on previous work by simultaneously taking into consideration (a) the importance of a latent host phenotype as an indicator of elevated risk of acquiring multiple infections, and (b) pathogen-pathogen relationships as an indicator of possible facilitation on one another. Our approach is informative but not intended as a substitute for controlled experiments or longitudinal studies in order to identify causal processes. Overall, our SEMs provide strong supporting evidence for a latent variable (in this case, a host phenotype) having a strong underlying influence on individual disease risk to multiple pathogens within our study system. We also identify novel pathogen covariance relationships potentially indicative of pathogen-facilitation, which warrant further investigation. We suggest mechanistic approaches such as structural equation modelling could be used more widely to understand complex cross-sectional information in a wide variety of disciplines.
